# Fatty Acid Metabolism in Carriers of Apolipoprotein E Epsilon 4 Allele: Is It Contributing to Higher Risk of Cognitive Decline and Coronary Heart Disease?

**DOI:** 10.3390/nu6104452

**Published:** 2014-10-20

**Authors:** Raphaël Chouinard-Watkins, Mélanie Plourde

**Affiliations:** Research Center on Aging, Health and Social Services Centre-University Institute of Geriatrics of Sherbrooke, Department of medicine, Université de Sherbrooke, 1036 Belvédère Sud, Sherbrooke, J1H 4C4, Canada; E-Mail: raphael.chouinard-watkins@usherbrooke.ca

**Keywords:** apolipoprotein E epsilon 4 allele, cognitive decline, coronary heart disease, docosahexaenoic acid, fatty acids

## Abstract

Apolipoprotein E (ApoE) is a protein playing a pivotal role in lipid homeostasis since it regulates cholesterol, triglyceride and phospholipid metabolism in the blood and the brain. APOE gene regulates the expression of this protein and has three different alleles: ε2, ε3 and ε4. Carrying an APOE4 allele is recognised as a genetic risk factor of late-onset Alzheimer’s disease (LOAD) and coronary heart disease (CHD). Consuming fatty fish, rich in long chain omega-3 fatty acids (LC omega-3), seems to be associated with risk reduction of developing LOAD and CHD but this link seems not to hold in APOE4 carriers, at least in LOAD. In CHD trials, APOE4 carriers supplemented with LC omega-3 were categorized as differential responders to the treatment with regards to CHD risk markers. This is potentially because fatty acid metabolism is disturbed in APOE4 carriers compared to the non-carriers. More specifically, homeostasis of LC omega-3 is disrupted in carriers of APOE4 allele and this is potentially because they β-oxidize more LC omega-3 than the non-carriers. Therefore, there is a potential shift in fatty acid selection for β-oxidation towards LC omega-3 which are usually highly preserved for incorporation into cell membranes.

## 1. Introduction

Apolipoprotein E (ApoE) is a 34 kDa protein with 299 amino acids and it was first identified as a component of triglycerides-rich lipoproteins. ApoE is located at the surface of chylomicrons, high density lipoproteins (HDL), intermediate density lipoproteins (IDL) and very low density lipoproteins (VLDL). Production of the ApoE protein is controlled by the APOE gene, for which three different alleles are recognized: ε2, ε3 and ε4 [1]. Therefore, there are three homozygous (APOE2/2, APOE3/3 and APOE4/4) and three heterozygous (APOE2/3, APOE2/4 and APOE3/4) polymorphisms of the APOE gene and the frequency of these polymorphisms vary greatly between populations [2,3]. In North America, those descending from the Europeans had an allele frequency as follows: APOE2: 7%–14%, APOE3: 74%–81% and APOE4: 11%–17% [2,3]. Expression of the ApoE protein isoforms differs by two amino acid substitutions at position 112 and/or 158. APOE2 has a cysteine at both positions, APOE3 has a cysteine-112 and an arginine-158 and APOE4 has an arginine at both positions [1,4].

ApoE plays a pivotal role in lipid homeostasis. It regulates cholesterol, triglyceride and phospholipid transport and metabolism via interactions with receptors of the LDL family (LDLr) [5]. ApoE plays a critical role on cholesterol catabolism when bounded to HDL via the cholesterol reverse transport system [5]. ApoE production occurs primarily in the liver and the brain and to a lesser extent, in macrophages [6,7]. ApoE within the brain plays a critical role in cholesterol and phospholipid transport to neurons. This process is likely mediated by activation of LDLr, which are highly expressed and distributed in neurons. ApoE does not cross the blood brain barrier [8], suggesting that there is no exchange between brain ApoE and ApoE within lipoproteins and other organs. Therefore, the brain seems to have its own pool of ApoE generated primarily by glial cells, in particular astrocytes which are also the main regulators of ApoE production [9].

Homozygous carriers of APOE4 have a 15-fold increased risk of late-onset Alzheimer’s disease (LOAD) as compared to the non-carriers [10,11]. It is unclear how APOE4 modulates LOAD pathology but neuropathological changes associated with LOAD such as β-amyloid (Aβ) plaque deposition occur as early as 30 years of age in APOE4 carriers [12]. Rate of brain atrophy in APOE4 carriers is accelerated compared to the non-carriers [13,14,15], potentially because APOE4 carriers have poor brain protection and poor brain repair mechanisms making this population more vulnerable to brain volume loss at a younger age [16,17]. The cognitive deficits, mostly on measures of delayed recall and spatial attention [18,19], are not confined to older APOE4 adults [20,21] and may also occur as early as midlife (35–40 years old), a decade or more before the onset of LOAD symptoms [22]. There is, therefore, a large window of action for environmental risk factors to modulate the clinical manifestation of LOAD.

APOE4 allele is also associated with an increased risk of developing cardiovascular-related complications. According to a meta-analysis published by Song *et al.* [23] in 2004, carrying at least one ε4 allele of APOE is associated with a 42% increased risk of developing coronary heart disease (CHD). On the other hand, one study reported that after controlling for LDL and HDL cholesterol, CHD risk was not associated with APOE genotype [24]. This suggests that increased CHD risk in APOE4 carriers seems to be attributed to disturbances in lipid homeostasis and most notably with regards to TG, cholesterol and LDL metabolism.

This review will focus on the current evidence on disturbed fatty acid metabolism in APOE4 carriers and whether this can contribute to their higher risk of developing cognitive decline and cardiovascular-related complications.

## 2. Fatty Acids Composition of the Human Brain and Heart

The brain is concentrated in long chain omega-3 fatty acids (LC omega-3), and more specifically in docosahexaenoic acid (DHA) which is a key molecule in neurotransmission, membrane repair and fluidity, cell signaling, initiation of anti-inflammatory processes and gene expression [25,26,27,28]. DHA is mainly obtained through fatty fish intake, which is positively correlated with higher plasma or erythrocyte DHA concentration [29,30]. In humans, synthesis of DHA from alpha-linolenic acid (ALA) is possible, albeit with a conversion rate less than 0.5% [31]. In animals, it appears that the brain may be able to synthesize limited amounts of DHA from ALA and EPA [32]. DHA consumption is thought to be protective against LOAD in animals via at least 12 neuroprotective effects, including limitation of the production and deposition of Aβ protein in the brain [33,34]. Hence, DHA appears to play pleiotropic effects on the central nervous system that may be protective against age-related and/or APOE4-related cognitive decline.

To our knowledge, fatty acid composition of the human heart has been analysed in at least three studies [35,36,37]. In the first study, fatty acid profiles were analysed in the phospholipid classes of the heart [35] whereas in the two others, fatty acid profiles were reported in total phospholipids. In the most recent study, participants were recruited on the basis of their low LC omega-3 consumption (<1 fish meal/week) [36]. In total phospholipids of the right atrial, total LC omega-3 and LC omega-6 fatty acids represented 30.3% of the total fatty acid content, with arachidonic acid being the most concentrated LC omega-6 and DHA the most concentrated LC omega-3 (20.8% and 4.8% of total fatty acids, respectively) [36]. Hence, even in the context of minimal DHA consumption, heart phospholipids appear to retain DHA. When participants were supplemented with 6 g/day EPA + DHA over 7, 14 or 21 days, EPA + DHA in the phospholipids were correlated with the duration of the supplementation and arachidonic acid content was inversely correlated with the duration of the supplementation. Despite similar DHA content in the erythrocyte of the control *vs.* the supplemented group, the latter had higher levels of DHA in heart phospholipids [36]. Hence, DHA is highly concentrated in the brain and the heart and modifying its turnover and kinetics could well be involved in the risk of developing LOAD and CHD.

## 3. LC Omega-3, Cognition and APOE4

The strongest evidence for a link between fish consumption and/or LC omega-3 intake and cognition stems from prospective epidemiological studies. At least ten such studies support the notion that higher fish intake is associated with lower risks of cognitive decline and LOAD [38,39,40,41,42,43,44,45,46,47,48]. Moreover, high erythrocyte LC omega-3 levels appear to be associated with better cognitive function in later life [49] along with a lower risk of cognitive decline [50,51,52,53,54]. Using a lipidomic approach, a recent paper reported a set of ten blood lipids associated to conversion to mild cognitive impairment [55] supporting that lipids can be important biomarkers of cognitive status. When adding APOE4 allele as a covariate in the statistical model, it was not statistically significant suggesting that the lipid biomarker panel was the same between carriers and non-carriers [55]. However, fish consumption in middle-aged individuals [41] has been associated with less occurrence of cognitive impairment later in life. The credibility of this association was strengthened by the publication of evidence that plasma DHA in the highest tertile is associated with a 65% reduced odds of all-caused dementia and that daily LC omega-3 supplement consumption was independently associated with a reduced risk of cognitive decline [56,57]. Despite general agreement amongst prospective epidemiological studies on the link between high fish intake and lower risk of cognitive decline, two prospective studies have reported that APOE4 carriers do not appear to be protected against dementia by a high fish-containing diet [39,42]. Moreover, in the most recent placebo-controlled study in LOAD patients [58], only those not carrying APOE4 and consuming the DHA-treatment had a decreased rate of cognitive change as compared to the placebo group. One potential confounding factor of this lack of association is an imbalance in the metabolism of LC omega-3 in APOE4 carriers since LC omega-3 concentration in erythrocytes is not correlated with better cognitive scores in both young (11-year-old) and older participants (65-year-old) carrying APOE4 [59] contrary to non-carriers. Hence, from epidemiological studies, it seems that individuals at higher risks of LOAD are those with less potential benefits from LC omega-3.

## 4. Prospective Studies on APOE4 and CHD

A meta-analysis on 37 retrospective and 11 prospective epidemiological studies with 15,492 cases of CHD and 32,965 controls reported that CHD odd ratio (OR) was 1.42 (1.26–1.61) in APOE4 carriers compared to homozygous carriers of APOE3 [23]. Although the statistics were strong, results from these 48 studies were highly heterogeneous, with OR for CHD ranging from 0.68 [60] to 4.1 [61] for APOE4 carriers. This is potentially explained by the inclusion/exclusion criterion of each study differing by age, diet and gender. A recent epidemiological study reported that in the older persons, the association between APOE allele and CHD seems inconsistent, even though APOE alleles clearly influence plasma LDL-C and may be linked to atherosclerosis [62].

When using stroke as the main outcome, a meta-analysis with 9027 cases of ischemic strokes and 61,730 controls showed that APOE3/4 and APOE4/4 carriers had higher OR for ischemic stroke and higher plasma levels of LDL-C compared with the non-carriers [63]. Hence, the authors hypothesised that higher risk of stroke could be mainly mediated by higher LDL-C levels in APOE4 carriers [63].

## 5. LC Omega-3, CHD and APOE4

Consumption of LC omega-3 is associated with a reduced risk of CHD in the general population [64,65,66]. In one study, there was a 14% lower risk of heart failure in participants consuming the highest quartile of LC omega-3 compared to the lowest quartile [64]. Higher fish and/or LC omega-3 intake were also associated with lower risk of cardiac sudden death and/or acute myocardial infarction [66,67].

To the best of our knowledge, there is no published prospective study that stratified by APOE allele to evaluate whether LC omega-3 consumption lowers the risk of CHD. It is increasingly recognized that LC omega-3 homeostasis changes with age [68,69] but also that there are interactions between age and APOE allele on fasting and postprandial lipid levels [70]. Moreover, LC omega-3 in the plasma is associated with the concentration of plasma lipoproteins in an APOE allele dependant manner [71]. Hence, defining whether higher risk of CHD in APOE4 carriers could be partly mediated by deregulation of LC omega-3 homeostasis is needed.

## 6. Clinical Trials with Dietary Interventions

### 6.1. Dietary Interventions with LC Omega-3 and Cognition

It can be argued that, since high DHA levels in the blood are linked with better cognition, individuals with cognitive decline would benefit from a DHA supplement, which would contribute in delaying the progression of such decline [72,73,74,75,76,77,78]. However, only the individuals with the mildest decline of cognition appear to benefit from an LC omega-3 intake compared with placebo [72,73,78]. Therefore, LC omega-3 are not therapeutically effective once LOAD is in more advanced stages but are rather molecules that contribute to the prevention of cognitive decline. Indeed, other studies in healthy adults and elderly show that LC omega-3 intake and/or higher LC omega-3 distribution in blood lipids tend towards better cognitive performance in verbal fluency, visuospatial skills and visual acuity. Increased plasma DHA levels have also been associated with a slower decline in working memory in APOE4 carriers only [79] while DHA intake improved attention scores in healthy elderly individuals carrying APOE4 [80] as compared with placebo. Hence, APOE4 carriers may benefit from an adequate duration and dose of LC omega-3 supplement. There is therefore a need for studying the kinetics of DHA in APOE4 carriers compared with the non-carriers.

### 6.2. Dietary Interventions with LC Omega-3 and CHD

Since higher plasma levels and/or intake of LC omega-3 are associated with lower risk of CHD in humans [64,65,66,81], taking a LC omega-3 supplement should therefore lower the risk of CHD. However, results from dietary intervention trials have been inconsistent [82,83]. The first trials analysing this hypothesis reported benefits on primary [84,85] or secondary [86,87] prevention of CHD events in the ones consuming a LC omega-3 supplement compared to no supplement [84,85,86] or compared to a placebo [87]. Moreover, a meta-analysis conducted in 2013 on 11 randomized, double-blind, placebo controlled trials reported similar results [88]. However, other recent studies reported that LC omega-3 supplementation brings no benefit to cardiovascular outcomes [89,90,91]. Nonetheless, it is important to note that the design, dose of LC omega-3, duration of follow-up and use of concomitant lipid lowering medications are potentially confounders bringing heterogeneity in the outcome measure of these studies. Therefore, the impact of consuming LC omega-3 on reducing the risk of CHD remains to date controversial and limit the use of LC omega-3 in the clinic.

With regards to the APOE4 allele, none of the aforementioned studies evaluated whether APOE allele changes the outcome. From our Pubmed screening, there was no study evaluating whether intake of LC omega-3 was effective in preventing CHD in carriers of APOE4 allele. There are, however, studies that evaluated levels of cardiovascular risk markers with regards to APOE allele and diet [70,92,93,94]. In one of these studies, the participants were supplemented with 3.7 g/day of DHA and 0.6 g/day of EPA for four weeks. There was an increased in total cholesterol and LDL-C in APOE4 carriers and this was mainly attributed to DHA [94]. These results support that, in APOE4 carriers, high doses of DHA may negate at some point the benefits of DHA in preventing CHD [94]. This study also support that metabolism of fatty acid in this population is misunderstood and deserve more attention for better prevention and treatment therapies. Adding to this observation, APOE4 carriers older than 50 years old had higher postprandial TG levels compared to the non-carriers when they were challenged with two tests meals [70]. Another study reported that APOE4 carriers have higher plasma levels of fasting TG and C-reactive protein in response to a sequential dietary intervention consisting of an eight-week low fat diet followed by eight weeks of high saturated fat diet to which a supplement of 3.45 g/day of DHA was added for the final eight weeks [92]. Hence, lipid metabolism seems to be disturbed in APOE4 carriers and this could contribute to their higher risk of developing CHD.

## 7. Fatty Acid Metabolism in APOE4 Carriers

Our recent findings have shown that, after consuming a diet containing 1.1 g/day of DHA for six weeks, the rise in DHA level was 60% lower in plasma TG of APOE4 carriers as compared to the non-carriers [95]. We, thereafter, investigated the kinetics of DHA using a single oral dose of 40 mg of uniformly carbon-13-labeled DHA (^13^C-DHA) before and during the last month of a LC omega-3 supplementation in carriers and non-carriers of APOE4. Before supplementation, mean concentration of ^13^C-DHA was 31% lower in plasma total lipids of APOE4 carriers compared to non-carriers during the 28-day post tracer intake [96]. These results are in line with our previously published results [95] and support transient lower DHA incorporation in plasma total lipids in APOE4 carriers prior to LC omega-3 supplementation. Before supplementation, cumulative β-oxidation 1-day to 28-day post tracer intake was higher in APOE4 carriers compared to the non-carriers [96]. While on the supplement, β-oxidation of ^13^C-DHA was 41%–70% lower in APOE4 carriers 1 h–8 h post tracer intake compared to the non-carriers but these numbers need to be validated since there were only four carriers of APOE4 [68]. Despite this low number, we can speculate that (1) intake of high doses of LC omega-3 in APOE4 carriers does not increase degradation through β-oxidation which is opposed to what we reported in the non-carriers [97], (2) DHA kinetics appear to be rebalanced in APOE4 carriers, at least for β-oxidation, supporting that an appropriate dose and duration of LC omega-3 could benefit this population.

More recently, we determined the fatty acid profile in fasted and postprandial lipoproteins within three triacylglycerol-rich lipoprotein (TRL) fractions: S_f_ > 400 (predominately chylomicron), S_f_ 60–400 (very low density lipoprotein 1, VLDL_1_), and S_f_ 20–60 (VLDL_2_) according to APOE genotype [98]. These analyses were performed in participants fed a high-fat, high saturated fat diet +3.45 g/day of docosahexaenoic acid (DHA) for eight weeks. We found that APOE4 carriers with low EPA- or DHA-status at fasting were potentially the ones having the most disrupted LC omega-3 metabolism after receiving a DHA supplement because EPA relative % at 5 h compared to 0 h (∆) was significantly reduced in APOE4 carriers from the low-EPA or -DHA group in the S_f_ > 400 fraction [98]. We also investigated the distribution of fatty acids within the high and low density lipoproteins (HDL and LDL) according to APOE genotype over a 28 days supplementation with LC omega-3 [99]. At baseline, the *n*-6/*n*-3 PUFA ratio in LDL was 17% higher in APOE4 carriers than non-carriers, but not in HDL. Linoleic acid in HDL was higher in APOE4 carriers than non-carriers, whereas palmitic acid in HDL and LDL and palmitoleic acid in LDL were lower in the carriers than the non-carriers over the 28 days supplementation [99].

Hence, in humans, there is increasing evidences supporting that fatty acid homeostasis is disturbed in APOE4 carriers compared to the non-carriers. Difference in fatty acid distribution in the lipoprotein is potentially associated with the lower blood ApoE concentration reported in APOE4 carriers than the non-carriers [100]. Moreover, APOE4 binds preferentially to VLDL and less to HDL when compared to APOE3 [100]. This explanation seems more valid than the one of higher affinity for LDLr since both isoforms bind to the LDLr with high affinity [101]. These mechanisms could have crucial implications on fatty acid uptake by hepatic cells, notably DHA, explaining why β-oxidation of DHA differs between carriers and non-carriers of APOE4 [96].

Since uptake of LC omega-3 by organs was not possible to investigate in humans, we used transgenic mice knock-in for human APOE4.

## 8. Animal Studies

Animal models are useful tools to investigate mechanisms responsible for the link between DHA intake and neuroprotection. APOE4 mice have memory decline similar to that reported in humans [102,103]; these declines are age-dependent [104] and deficits are concomitant with hippocampal and amygdala dysfunctions [105,106]. ApoE-containing particles act as ligands for LDL-receptor family members and play critical roles in maintaining brain lipid homeostasis and associated synaptic and neuronal integrity [5,107,108,109]. Recent evidences support that BBB permeability is higher in APOE4-knock-in mice than in APOE3 knock-in mice [106,107]. One of the best methodological approach to assess brain uptake and permeability of DHA is *in situ* intracerebral perfusion adapted for the mouse [110,111] because DHA is directly infused into the carotid artery [110,111] and thus bypasses the peripheral blood circulation. This technique can assess whether imbalances in LC omega-3 metabolism that occur during aging and in APOE4 carriers is leading to dysfunctional uptake of DHA by the brain. We recently tested this hypothesis in 4-month-old mice and in 13-month-old mice homozygous for APOE4, APOE3 or APOE2 allele [112]. At 4 months and 13 months of age, ^14^C-DHA brain uptake was 18% and 24% lower in mice carrying the APOE4 genotype compared to mice carrying APOE2 genotype. In plasma total lipids, there was no genotype effect for DHA in the 4-month-old mice, whereas, in the 13-month-old mice, APOE4 mice had 34% higher % DHA compared to APOE2 mice. In frontal cortex, % DHA was lower in 13-month-old mice compared to 4-month-old mice with the same genotype. Moreover, at 13 months, APOE4 mice had 9% lower % DHA than APOE2 mice. As reported in humans [113], ApoE protein levels in APOE4 mice of 4 and 13 months were significantly lower compared to other APOE genotype. APOE2 mice aged 13 months had significantly higher ApoE protein levels compared to both 4-month-old mice and 13-month-old APOE4 mice [112]. In the same mouse model, we sought to determine if APOE genotype modulates expression of key fatty acid handling proteins, thereby disrupting transport and uptake of fatty acids by the liver and the adipose tissue.

LC omega-3, alpha-linolenic acid and DHA concentrations in the adipose tissue and the liver of APOE4 mice were significantly lower than the APOE3 mice. However, the fatty acid transport proteins of the adipose tissue and the liver (*i.e.*, FATP1 and FATP5), together with the liver fatty acid binding protein FABP1, were higher in APOE4 carriers, suggesting higher capacity for fatty acid uptake by the cells [114]. This disconnect between the level of fatty acids in the tissues and the plasma and their capacity for uptake support that FATPs and FABPs cellular regulations are modulated by APOE genotype. Moreover, carnitine palmitoyltransferase 1 (CPT1) levels were ~25% higher in APOE4 mice suggesting higher capacity for fatty acid to enter mitochondria for β-oxidation since this is the rate limiting-enzyme of this metabolic pathway [114].

Altogether, these results support our hypothesis that the expression of APOE4 leads to important modifications and imbalances in the metabolism and the kinetics of LC omega-3 in APOE4 carriers.

## 9. Overlap between Cognitive Decline, Load and CHD

LOAD and vascular dementia, the second most frequent type of dementia, were originally distinguished on the basis that vascular pathology was not the main underlying culprit of most dementia [115]. On the other hand, there is increasing evidence suggesting that LOAD may have a more important vascular component than originally thought [115]. Indeed, patients with LOAD often present reduced cerebral blood flow [116] together with white matter abnormality, microvascular degeneration and other vascular pathology [117]. Moreover, risk factors for both LOAD and CHD are strikingly similar since more than a third of LOAD cases worldwide may be attributable to seven modifiable risk factors [118]. Of these, at least five are associated with CHD: diabetes, hypertension, obesity, physical inactivity and smoking. Some studies also reported that hypercholesterolemia may increase the risk for LOAD [119,120].

One hypothesis for the vascular component of LOAD involves the BBB integrity, which has been reported to be compromised during cognitive decline. Interestingly, animal studies suggest that ApoE plays a role on vascular integrity and that a lack of ApoE leads to BBB breakdown via a cyclophilin A related proinflammatory pathway in the pericytes [121]. Furthermore, this pathway also appears to explain why APOE4 mice have higher BBB permeability than APOE3 mice [121]. In humans, old APOE4 carriers also have elevated markers of BBB impairment when compared to young APOE4 carriers or age matched non-carriers [122].

Hence, these findings suggest that the link between cognitive decline and vascular pathology is present and that APOE genotype may contribute to this association.

## 10. Does Fatty Acid Metabolism Disruption Contributes to Higher Risk of Cognitive Decline and CHD in APOE4 Carriers?

Over the last four years, our research group search to better understand the disturbed fatty acid metabolism in APOE4 carriers and whether this could contribute to higher risk of cognitive decline.

**Figure 1:**
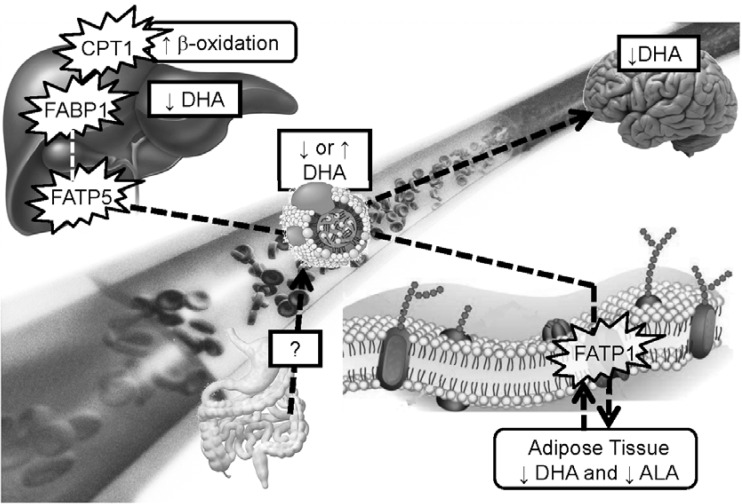
Working hypothesis for explaining how disrupt docosahexaenoic acid (DHA) kinetics in apolipoprotein E epsilon 4 (APOE4) carriers could be involved in the risk of cognitive decline and coronary heart disease. Blood DHA level reflects the balance between the uptake and release of fatty acids from organs, such as the liver and the adipose tissue. In humans and animals carrying an APOE4 allele, blood DHA was sometimes higher and some other times lower compared to the non-carriers. In mice knock-in for human APOE4 allele, adipose tissue and liver fatty acid transport protein (FATP) were unregulated compared to APOE3 mice. Hence, we would anticipate higher levels of DHA in adipose tissue and liver but it was the opposite, displaying lower levels of DHA in both tissues. In the liver, fatty acid binding protein (FABP) together with carnitine palmitoyl transferase 1 (CPT1) were unregulated in APOE4 mice compared to APOE3 mice. Hence, this shows higher capacity for β-oxidation of fatty acids in APOE4 carriers compared to the non-carriers. Brain DHA uptake was also lower in 4-month- and 13-month-old APOE4 mice compared to APOE2 mice and lower levels of DHA in the brain membranes were reported in 13-month-old APOE4 mice only [112]. In humans, it seems that postprandial DHA was lower in APOE4 carriers compared to the non-carriers. Hence, the gut-to-brain connection may play an important role in the delivery of LC omega-3 in APOE4 carriers for better brain and heart health.

In humans, plasma DHA levels were not consistent among the studies stratifying by APOE allele. There are different reasons explaining this discrepancy. First, the lipid class in which dosages are performed is not uniform; sometimes DHA was dosed in total lipids, phospholipids or red blood cells. In the first study reporting a gene-by-diet interaction, we have shown that it was specific to triglycerides and free fatty acid classes supporting that this interaction is probably specific to lipid classes [95]. Since there is a lack of reference range for individual fatty acids, it is not currently possible to establish whether there are disease-associated risks of DHA deficiency in the blood. Moreover, there are other factors affecting the range of DHA levels in the blood such as aging [69,123,124] and potentially body mass index [97]. Our results with APOE4 mice are similar to what we reported in humans and support that APOE4-modification in DHA homeostasis could alter proteins levels involved in the handling of fatty acids. These changes are summarized in Figure 1.

Our thoughts are now oriented towards studying whether fatty acid selection for peroxisomal and/or mitochondrial β-oxidation could be involved in the link between DHA homeostasis and risk of cognitive decline and CHD. Peroxisomes are involved in reactive oxygen species generation and removal together with the first step of β-oxidation of long chain fatty acids such as DHA. One recent study showed that peroxisome may be a first line of defence in support to mitochondria and this is potentially throughout fatty acyl β-oxidation, likely providing mitochondria with acetyl-CoA and shortened acyl-CoA [125]. Using an inhibitor of peroxisomal β-oxidation, it was shown that very long chain fatty acids accumulated in the brain together with higher accumulation of β-amyloid, a protein accumulating in the aging brain, particularly in those suffering from LOAD [126]. In humans, there are evidences that some of the enzymes involved in liver fatty acid β-oxidation are deregulated in patients with LOAD [127] and CPT1 activity seems to be lower in the brain [128]. With regards to the heart, fatty acid β-oxidation provides energy for myocyte survival and also regulates cardiac TG homeostasis by preventing TG accumulation [129]. Myocardial total CPT appears to be deficient in patients with CHD [130] thereby impairing β-oxidation. Whether this could lead to disturbed LC omega-3 in the heart remains to be established. Additionally, the role of APOE genotype on cardiac and brain fatty acid β-oxidation needs to be investigated.

Altogether, these results suggest that β-oxidation potentially plays a more crucial role than expected in the process of LOAD and CHD and this needs further investigations in humans and animals. Other questions important to be answered are why the presence of APOE4 is associated with a loss of BBB integrity during aging and whether this could also contribute to disrupt DHA homeostasis. A novel transporter named major facilitator super family domain containing 2a (Mfsd2a) has recently been reported to play a crucial role in BBB formation [131] and a deletion of this transporter in mice (Mfsd2a^−/−^) resulted in BBB leakage with no alteration of the BBB vascular network [131]. Interestingly, the brain of the Mfsd2a^−/−^ mice was also deficient in DHA [132]. Moreover, by performing brain transport assay with carbon 14 lysophosphatidylcholine (LPC) DHA and oleic acid, the authors showed that Mfsd2a is crucial in the transport of these fatty acids in the LPC form and seems to be an important route by which DHA enters the brain [132]. This suggests a dual role of Mfsd2a for BBB function and brain DHA uptake [133,134] and provide a better understanding of brain DHA homeostasis. Whether APOE genotype plays a role on the function of this transporter remains to be evaluated.

## 11. Conclusions

In this paper, we highlighted that people carrying at least one allele of APOE4 seems to have a deregulated fatty acid metabolism with emphasis on disrupted DHA homeostasis. To date, it is not clear how this could play a role in the risk of developing LOAD and/or CHD but it could involve the following processes.

1. Shift in fatty acid selection for β-oxidation where DHA becomes highly β-oxidized in APOE4 carriers whereas in the non-carriers, DHA is highly conserved.
2. In APOE4 carriers, brain uptake of DHA seems lower resulting in lower brain membrane DHA over time. This could play a role in neurotransmission and expression of genes and proteins involved in brain health but this needs further investigation.
3. APOE4 carriers respond differently than non-carriers to dietary interventions involving lipids such that modulating lipoprotein levels may include managing fatty acid circulating in the blood. Providing higher doses of LC omega-3 to this population could be necessary to obtain a similar response compared to the non-carriers supplemented with lower doses of LC omega-3.

## References

[1] Weisgraber K.H., Rall S.C., Mahley R.W. (1981). Human E apoprotein heterogeneity. Cysteine-arginine interchanges in the amino acid sequence of the Apo-E isoforms. J. Biol. Chem..

[2] Garenc C., Aubert S., Laroche J., Girouard J., Vohl M.C., Bergeron J., Rousseau F., Julien P. (2004). Population prevalence of APOE, APOC3 and PPAR-alpha mutations associated to hypertriglyceridemia in French Canadians. J. Hum. Genet..

[3] Bullido M.J., Artiga M.J., Recuero M., Sastre I., Garcia M.A., Aldudo J., Lendon C., Han S.W., Morris J.C., Frank A. (1998). A polymorphism in the regulatory region of APOE associated with risk for Alzheimer’s dementia. Nat. Genet..

[4] Rall S.C., Weisgraber K.H., Mahley R.W. (1982). Human apolipoprotein E. The complete amino acid sequence. J. Biol. Chem..

[5] Mahley R.W. (1988). Apolipoprotein E: Cholesterol transport protein with expanding role in cell biology. Science.

[6] Lin C.T., Xu Y.F., Wu J.Y., Chan L. (1986). Immunoreactive apolipoprotein E is a widely distributed cellular protein. Immunohistochemical localization of apolipoprotein E in baboon tissues. J. Clin. Investig..

[7] Elshourbagy N.A., Liao W.S., Mahley R.W., Taylor J.M. (1985). Apolipoprotein E mRNA is abundant in the brain and adrenals, as well as in the liver, and is present in other peripheral tissues of rats and marmosets. Proc. Natl. Acad. Sci. USA.

[8] Liu M., Kuhel D.G., Shen L., Hui D.Y., Woods S.C. (2012). Apolipoprotein E does not cross the blood-cerebrospinal fluid barrier, as revealed by an improved technique for sampling CSF from mice. Am. J. Physiol. Regul. Integr. Comp. Physiol..

[9] Pitas R.E., Boyles J.K., Lee S.H., Foss D., Mahley R.W. (1987). Astrocytes synthesize apolipoprotein E and metabolize apolipoprotein E-containing lipoproteins. Biochim. Biophys. Acta.

[10] Farrer L.A.P., Cupples L.A.P., Haines J.L.P., Hyman B.M.D.P., Kukull W.A.P., Mayeux R.M.D., Myers R.H.P., Pericak-Vance M.A.P., Risch N.P., van Duijn C.M.P. (1997). Effects of age, sex, and ethnicity on the association between apolipoprotein E genotype and Alzheimer disease: A meta-analysis. JAMA.

[11] Coon K.D., Myers A.J., Craig D.W., Webster J.A., Pearson J.V., Lince D.H., Zismann V.L., Beach T.G., Leung D., Bryden L. (2007). A high-density whole-genome association study reveals that APOE is the major susceptibility gene for sporadic late-onset Alzheimer’s disease. J. Clin. Psychiatry.

[12] Kok E., Haikonen S., Luoto T., Huhtala H., Goebeler S., Haapasalo H., Karhunen P.J. (2009). Apolipoprotein E-dependent accumulation of Alzheimer disease-related lesions begins in middle age. Ann. Neurol..

[13] Filippini N., Zarei M., Beckmann C.F., Galluzzi S., Borsci G., Testa C., Bonetti M., Beltramello A., Ghidoni R., Benussi L. (2009). Regional atrophy of transcallosal prefrontal connections in cognitively normal APOE epsilon4 carriers. J. Magn. Reson. Imaging.

[14] Jak A.J., Houston W.S., Nagel B.J., Corey-Bloom J., Bondi M.W. (2007). Differential cross-sectional and longitudinal impact of APOE genotype on hippocampal volumes in nondemented older adults. Dement. Geriatr. Cogn. Disord..

[15] Chen K., Reiman E.M., Alexander G.E., Caselli R.J., Gerkin R., Bandy D., Domb A., Osborne D., Fox N., Crum W.R. (2007). Correlations between apolipoprotein E epsilon4 gene dose and whole brain atrophy rates. Am. J. Psychiatry.

[16] Laitinen M.H., Ngandu T., Rovio S., Helkala E.L., Uusitalo U., Viitanen M., Nissinen A., Tuomilehto J., Soininen H., Kivipelto M. (2006). Fat intake at midlife and risk of dementia and Alzheimer’s disease: A population-based study. Dement. Geriatr. Cogn. Disord..

[17] Luchsinger J.A., Tang M.X., Shea S., Mayeux R. (2002). Caloric intake and the risk of Alzheimer disease. Arch Neurol..

[18] Bondi M.W., Salmon D.P., Galasko D., Thomas R.G., Thal L.J. (1999). Neuropsychological function and apolipoprotein E genotype in the preclinical detection of Alzheimer’s disease. Psychol. Aging.

[19] Greenwood P.M., Lambert C., Sunderland T., Parasuraman R. (2005). Effects of apolipoprotein E genotype on spatial attention, working memory, and their interaction in healthy, middle-aged adults: Results from the national institute of mental health’s biocard study. Neuropsychology.

[20] Baxter L.C., Caselli R.J., Johnson S.C., Reiman E., Osborne D. (2003). Apolipoprotein E epsilon 4 affects new learning in cognitively normal individuals at risk for Alzheimer’s disease. Neurobiol. Aging.

[21] Scarmeas N., Habeck C.G., Hilton J., Anderson K.E., Flynn J., Park A., Stern Y. (2005). APOE related alterations in cerebral activation even at college age. J. Neurol. Neurosurg. Psychiatry.

[22] Greenwood P.M., Sunderland T., Putnam K., Levy J., Parasuraman R. (2005). Scaling of visuospatial attention undergoes differential longitudinal change as a function of APOE genotype prior to old age: Results from the NIMH BIOCARD study. Neuropsychology.

[23] Song Y., Stampfer M.J., Liu S. (2004). Meta-analysis: Apolipoprotein E genotypes and risk for coronary heart disease. Ann. Intern. Med..

[24] Ward H., Mitrou P.N., Bowman R., Luben R., Wareham N.J., Khaw K.T., Bingham S. (2009). APOE genotype, lipids, and coronary heart disease risk: A prospective population study. Arch. Intern. Med..

[25] Alessandri J.M., Guesnet P., Vancassel S., Astorg P., Denis I., Langelier B., Aid S., Poumes-Ballihaut C., Champeil-Potokar G., Lavialle M. (2004). Polyunsaturated fatty acids in the central nervous system: Evolution of concepts and nutritional implications throughout life. Reprod. Nutr. Dev..

[26] Calon F., Lim G.P., Yang F., Morihara T., Teter B., Ubeda O., Rostaing P., Triller A., Salem N., Ashe K.H. (2004). Docosahexaenoic acid protects from dendritic pathology in an Alzheimer’s disease mouse model. Neuron.

[27] Jump D.B., Botolin D., Wang Y., Xu J., Christian B., Demeure O. (2005). Fatty acid regulation of hepatic gene transcription. J. Nutr..

[28] Bouwens M., van de Rest O., Dellschaft N., Bromhaar M.G., de Groot L.C., Geleijnse J.M., Muller M., Afman L.A. (2009). Fish-oil supplementation induces antiinflammatory gene expression profiles in human blood mononuclear cells. Am. J. Clin. Nutr..

[29] Arterburn L.M., Hall E.B., Oken H. (2006). Distribution, interconversion, and dose response of *n*-3 fatty acids in humans. Am. J. Clin. Nutr..

[30] Vidgren H.M., Agren J.J., Schwab U., Rissanen T., Hanninen O., Uusitupa M.I. (1997). Incorporation of *n*-3 fatty acids into plasma lipid fractions, and erythrocyte membranes and platelets during dietary supplementation with fish, fish oil, and docosahexaenoic acid-rich oil among healthy young men. Lipids.

[31] Plourde M., Cunnane S.C. (2007). Extremely limited synthesis of long chain polyunsaturates in adults: Implications for their dietary essentiality and use as suppements. Appl. Physiol. Nutr. Metab..

[32] Bernoud N., Fenart L., Benistant C., Pageaux J.F., Dehouck M.P., Moliere P., Lagarde M., Cecchelli R., Lecerf J. (1998). Astrocytes are mainly responsible for the polyunsaturated fatty acid enrichment in blood-brain barrier endothelial cells *in vitro*. J. Lipid Res..

[33] Boudrault C., Bazinet R.P., Ma D.W. (2009). Experimental models and mechanisms underlying the protective effects of *n*-3 polyunsaturated fatty acids in Alzheimer’s disease. J. Nutr. Biochem..

[34] Cole G.M., Ma Q.L., Frautschy S.A. (2010). Dietary fatty acids and the aging brain. Nutr. Rev..

[35] Rocquelin G., Guenot L., Astorg P.O., David M. (1989). Phospholipid content and fatty acid composition of human heart. Lipids.

[36] Metcalf R.G., James M.J., Gibson R.A., Edwards J.R., Stubberfield J., Stuklis R., Roberts-Thomson K., Young G.D., Cleland L.G. (2007). Effects of fish-oil supplementation on myocardial fatty acids in humans. Am. J. Clin. Nutr..

[37] Rocquelin G., Guenot L., Justrabo E., Grynberg A., David M. (1985). Fatty acid composition of human heart phospholipids: Data from 53 biopsy specimens. J. Mol. Cell. Cardiol..

[38] Barberger-Gateau P., Letenneur L., Deschamps V., Peres K., Dartigues J.F., Renaud S. (2002). Fish, meat, and risk of dementia: Cohort study. BMJ.

[39] Barberger-Gateau P., Raffaitin C., Letenneur L., Berr C., Tzourio C., Dartigues J.F., Alperovitch A. (2007). Dietary patterns and risk of dementia: The three-city cohort study. Neurology.

[40] Beydoun M.A., Kaufman J.S., Sloane P.D., Heiss G., Ibrahim J. (2008). *n*-3 Fatty acids, hypertension and risk of cognitive decline among older adults in the atherosclerosis risk in communities (ARIC) study. Public Health Nutr..

[41] Eskelinen M.H., Ngandu T., Helkala E.L., Tuomilehto J., Nissinen A., Soininen H., Kivipelto M. (2008). Fat intake at midlife and cognitive impairment later in life: A population-based CAIDE study. Int. J. Geriatr. Psychiatry.

[42] Huang T.L., Zandi P.P., Tucker K.L., Fitzpatrick A.L., Kuller L.H., Fried L.P., Burke G.L., Carlson M.C. (2005). Benefits of fatty fish on dementia risk are stronger for those without APOE epsilon4. Neurology.

[43] Kalmijn S., Feskens E.J., Launer L.J., Kromhout D. (1997). Polyunsaturated fatty acids, antioxidants, and cognitive function in very old men. Am. J. Epidemiol..

[44] Kalmijn S., Launer L.J., Ott A., Witteman J.C., Hofman A., Breteler M.M. (1997). Dietary fat intake and the risk of incident dementia in the Rotterdam Study. Ann. Neurol..

[45] Morris M.C., Evans D.A., Bienias J.L., Tangney C.C., Bennett D.A., Wilson R.S., Aggarwal N., Schneider J. (2003). Consumption of fish and *n*-3 fatty acids and risk of incident Alzheimer disease. Arch. Neurol..

[46] Morris M.C., Evans D.A., Tangney C.C., Bienias J.L., Wilson R.S. (2005). Fish consumption and cognitive decline with age in a large community study. Arch. Neurol..

[47] Van Gelder B.M., Tijhuis M., Kalmijn S., Kromhout D. (2007). Fish consumption, *n*-3 fatty acids, and subsequent 5-y cognitive decline in elderly men: The Zutphen elderly study. Am. J. Clin. Nutr..

[48] Vercambre M.N., Boutron-Ruault M.C., Ritchie K., Clavel-Chapelon F., Berr C. (2009). Long-term association of food and nutrient intakes with cognitive and functional decline: A 13-year follow-up study of elderly French women. Br. J. Nutr..

[49] Whalley L.J., Fox H.C., Wahle K.W., Starr J.M., Deary I.J. (2004). Cognitive aging, childhood intelligence, and the use of food supplements: Possible involvement of *n*-3 fatty acids. Am. J. Clin. Nutr..

[50] Beydoun M.A., Kaufman J.S., Satia J.A., Rosamond W., Folsom A.R. (2007). Plasma *n*-3 fatty acids and the risk of cognitive decline in older adults: The atherosclerosis risk in communities study. Am. J. Clin. Nutr..

[51] Dullemeijer C., Durga J., Brouwer I.A., van de Rest O., Kok F.J., Brummer R.J., van Boxtel M.P., Verhoef P. (2007). *n*-3 Fatty acid proportions in plasma and cognitive performance in older adults. Am. J. Clin. Nutr..

[52] Heude B., Ducimetiere P., Berr C. (2003). Cognitive decline and fatty acid composition of erythrocyte membranes—The EVA study. Am. J. Clin. Nutr..

[53] Samieri C., Feart C., Letenneur L., Dartigues J.F., Peres K., Auriacombe S., Peuchant E., Delcourt C., Barberger-Gateau P. (2008). Low plasma eicosapentaenoic acid and depressive symptomatology are independent predictors of dementia risk. Am. J. Clin. Nutr..

[54] Schaefer E.J., Bongard V., Beiser A.S., Lamon-Fava S., Robins S.J., Au R., Tucker K.L., Kyle D.J., Wilson P.W., Wolf P.A. (2006). Plasma phosphatidylcholine docosahexaenoic acid content and risk of dementia and Alzheimer disease: The Framingham Heart Study. Arch. Neurol..

[55] Mapstone M., Cheema A.K., Fiandaca M.S., Zhong X., Mhyre T.R., MacArthur L.H., Hall W.J., Fisher S.G., Peterson D.R., Haley J.M. (2014). Plasma phospholipids identify antecedent memory impairment in older adults. Nat. Med..

[56] Ng T.P., Gao Q., Niti M., Feng L., Yap K.B. (2011). Omega-3 polyunsaturated fatty acid supplements and cognitive decline: Singapore Longitudinal Aging Studies. J. Nutr. Health Aging.

[57] Kritz-Silverstein D., Lopez L.B., Barrett Connor E. (2011). High dietary and plasma levels of the omega-3 fatty acid docosahexaenoic acid are associated with decreased dementia risk: The Rancho Bernardo study. J. Nutr. Health Aging.

[58] Quinn J.F., Raman R., Thomas R.G., Yurko-Mauro K., Nelson E.B., Van Dyck C., Galvin J.E., Emond J., Jack C.R., Weiner M. (2010). Docosahexaenoic acid supplementation and cognitive decline in Alzheimer disease: A randomized trial. JAMA.

[59] Whalley L.J., Deary I.J., Starr J.M., Wahle K.W., Rance K.A., Bourne V.J., Fox H.C. (2008). *n*-3 Fatty acid erythrocyte membrane content, APOE varepsilon4, and cognitive variation: An observational follow-up study in late adulthood. Am. J. Clin. Nutr..

[60] Stuyt P.M., Brenninkmeijer B.J., Demacker P.N., Hendriks J.C., van Elteren P., Stalenhoef A.F., van’t Laar A. (1991). Apolipoprotein E phenotypes, serum lipoproteins and apolipoproteins in angiographically assessed coronary heart disease. Scand. J. Clin. Lab. Investig..

[61] Salazar L.A., Hirata M.H., Giannini S.D., Forti N., Diament J., Lima T.M., Hirata R.D. (2000). Seven DNA polymorphisms at the candidate genes of atherosclerosis in Brazilian women with angiographically documented coronary artery disease. Clin. Chim. Acta.

[62] Haan M.N., Mayeda E.R. (2010). Apolipoprotein E genotype and cardiovascular diseases in the elderly. Curr. Cardiovasc. Risk Rep..

[63] Khan T.A., Shah T., Prieto D., Zhang W., Price J., Fowkes G.R., Cooper J., Talmud P.J., Humphries S.E., Sundstrom J. (2013). Apolipoprotein E genotype, cardiovascular biomarkers and risk of stroke: Systematic review and meta-analysis of 14,015 stroke cases and pooled analysis of primary biomarker data from up to 60,883 individuals. Int. J. Epidemiol..

[64] Djousse L., Akinkuolie A.O., Wu J.H., Ding E.L., Gaziano J.M. (2012). Fish consumption, omega-3 fatty acids and risk of heart failure: A meta-analysis. Clin. Nutr..

[65] Siscovick D.S., Raghunathan T.E., King I., Weinmann S., Wicklund K.G., Albright J., Bovbjerg V., Arbogast P., Smith H., Kushi L.H. (1995). Dietary intake and cell membrane levels of long-chain *n*-3 polyunsaturated fatty acids and the risk of primary cardiac arrest. JAMA.

[66] Tavani A., Pelucchi C., Negri E., Bertuzzi M., la Vecchia C. (2001). *n*-3 Polyunsaturated fatty acids, fish, and nonfatal acute myocardial infarction. Circulation.

[67] Albert C.M., Hennekens C.H., O’Donnell C.J., Ajani U.A., Carey V.J., Willett W.C., Ruskin J.N., Manson J.E. (1998). Fish consumption and risk of sudden cardiac death. JAMA.

[68] Hennebelle M., Plourde M., Chouinard-Watkins R., Castellano C.A., Barberger-Gateau P., Cunnane S.C. (2014). Ageing and APOE change DHA homeostasis: Relevance to age-related cognitive decline. Proc. Nutr. Soc..

[69] Fortier M., Tremblay-Mercier J., Plourde M., Chouinard-Watkins R., Vandal M., Pifferi F., Freemantle E., Cunnane S.C. (2010). Higher plasma *n*-3 fatty acid status in the moderately healthy elderly in southern Quebec: Higher fish intake or aging-related change in *n*-3 fatty acid metabolism?. Prostaglandins Leukot. Essent. Fatty Acids.

[70] Carvalho-Wells A.L., Jackson K.G., Gill R., Olano-Martin E., Lovegrove J.A., Williams C.M., Minihane A.M. (2010). Interactions between age and APOE genotype on fasting and postprandial triglycerides levels. Atherosclerosis.

[71] Liang S., Steffen L.M., Steffen B.T., Guan W., Weir N.L., Rich S.S., Manichaikul A., Vargas J.D., Tsai M.Y. (2013). APOE genotype modifies the association between plasma omega-3 fatty acids and plasma lipids in the Multi-Ethnic Study of Atherosclerosis (MESA). Atherosclerosis.

[72] Chiu C.C., Su K.P., Cheng T.C., Liu H.C., Chang C.J., Dewey M.E., Stewart R., Huang S.Y. (2008). The effects of omega-3 fatty acids monotherapy in Alzheimer’s disease and mild cognitive impairment: A preliminary randomized double-blind placebo-controlled study. Prog. Neuropsychopharmacol. Biol. Psychiatry.

[73] Freund-Levi Y., Eriksdotter-Jonhagen M., Cederholm T., Basun H., Faxen-Irving G., Garlind A., Vedin I., Vessby B., Wahlund L.O., Palmblad J. (2006). Omega-3 fatty acid treatment in 174 patients with mild to moderate Alzheimer disease: OmegAD study: A randomized double-blind trial. Arch. Neurol..

[74] Kotani S., Sakaguchi E., Warashina S., Matsukawa N., Ishikura Y., Kiso Y., Sakakibara M., Yoshimoto T., Guo J., Yamashima T. (2006). Dietary supplementation of arachidonic and docosahexaenoic acids improves cognitive dysfunction. Neurosci. Res..

[75] Suzuki H., Morikawa Y., Takahashi H. (2001). Effect of DHA oil supplementation on intelligence and visual acuity in the elderly. World Rev. Nutr. Diet..

[76] Terano T., Fujishiro S., Ban T., Yamamoto K., Tanaka T., Noguchi Y., Tamura Y., Yazawa K., Hirayama T. (1999). Docosahexaenoic acid supplementation improves the moderately severe dementia from thrombotic cerebrovascular diseases. Lipids.

[77] Scheltens P., Kamphuis P.J., Verhey F.R., Olde Rikkert M.G., Wurtman R.J., Wilkinson D., Twisk J.W., Kurz A. (2010). Efficacy of a medical food in mild Alzheimer’s disease: A randomized, controlled trial. Alzheimers Dement..

[78] Yurko-Mauro K., McCarthy D., Rom D., Nelson E.B., Ryan A.S., Blackwell A., Salem N., Stedman M. (2010). Beneficial effects of docosahexaenoic acid on cognition in age-related cognitive decline. Alzheimers Dement..

[79] Samieri C., Feart C., Proust-Lima C., Peuchant E., Dartigues J.F., Amieva H., Barberger-Gateau P. (2011). Omega-3 fatty acids and cognitive decline: Modulation by apoeepsilon4 allele and depression. Neurobiol. Aging.

[80] Van de Rest O., Geleijnse J.M., Kok F.J., van Staveren W.A., Dullemeijer C., Olderikkert M.G., Beekman A.T., de Groot C.P. (2008). Effect of fish oil on cognitive performance in older subjects: A randomized, controlled trial. Neurology.

[81] Albert C.M., Campos H., Stampfer M.J., Ridker P.M., Manson J.E., Willett W.C., Ma J. (2002). Blood levels of long-chain *n*-3 fatty acids and the risk of sudden death. N. Engl. J. Med..

[82] Calder P.C., Yaqoob P. (2012). Marine omega-3 fatty acids and coronary heart disease. Curr. Opin. Cardiol..

[83] Kromhout D., Yasuda S., Geleijnse J.M., Shimokawa H. (2012). Fish oil and omega-3 fatty acids in cardiovascular disease: Do they really work?. Eur. Heart J..

[84] Oikawa S., Yokoyama M., Origasa H., Matsuzaki M., Matsuzawa Y., Saito Y., Ishikawa Y., Sasaki J., Hishida H., Itakura H. (2009). Suppressive effect of EPA on the incidence of coronary events in hypercholesterolemia with impaired glucose metabolism: Sub-analysis of the Japan EPA Lipid Intervention Study (JELIS). Atherosclerosis.

[85] Yokoyama M., Origasa H., Matsuzaki M., Matsuzawa Y., Saito Y., Ishikawa Y., Oikawa S., Sasaki J., Hishida H., Itakura H. (2007). Effects of eicosapentaenoic acid on major coronary events in hypercholesterolaemic patients (JELIS): A randomised open-label, blinded endpoint analysis. Lancet.

[86] GISSI-Prevenzione Investigators (Gruppo Italiano per lo Studio della Sopravvivenza nell’Infarto miocardico) (1999). Dietary supplementation with *n*-3 polyunsaturated fatty acids and vitamin E after myocardial infarction: Results of the GISSI-Prevenzione trial. Lancet.

[87] Gissi H.F.I., Tavazzi L., Maggioni A.P., Marchioli R., Barlera S., Franzosi M.G., Latini R., Lucci D., Nicolosi G.L., Porcu M. (2008). Effect of *n*-3 polyunsaturated fatty acids in patients with chronic heart failure (the GISSI-HF trial): A randomised, double-blind, placebo-controlled trial. Lancet.

[88] Casula M., Soranna D., Catapano A.L., Corrao G. (2013). Long-term effect of high dose omega-3 fatty acid supplementation for secondary prevention of cardiovascular outcomes: A meta-analysis of randomized, placebo controlled trials [corrected]. Atheroscler. Suppl..

[89] Kromhout D., Giltay E.J., Geleijnse J.M., Alpha Omega Trial Group (2010). *n*-3 Fatty acids and cardiovascular events after myocardial infarction. N. Engl. J. Med..

[90] Galan P., Kesse-Guyot E., Czernichow S., Briancon S., Blacher J., Hercberg S., SU.FOL.OM3 Collaborative Group (2010). Effects of B vitamins and omega 3 fatty acids on cardiovascular diseases: A randomised placebo controlled trial. BMJ.

[91] Rauch B., Schiele R., Schneider S., Diller F., Victor N., Gohlke H., Gottwik M., Steinbeck G., del Castillo U., Sack R. (2010). Omega, a randomized, placebo-controlled trial to test the effect of highly purified omega-3 fatty acids on top of modern guideline-adjusted therapy after myocardial infarction. Circulation.

[92] Carvalho-Wells A.L., Jackson K.G., Lockyer S., Lovegrove J.A., Minihane A.M. (2012). APOE genotype influences triglyceride and C-reactive protein responses to altered dietary fat intake in UK adults. Am. J. Clin. Nutr..

[93] Minihane A.M., Khan S., Leigh-Firbank E.C., Talmud P., Wright J.W., Murphy M.C., Griffin B.A., Williams C.M. (2000). APOE polymorphism and fish oil supplementation in subjects with an atherogenic lipoprotein phenotype. Arterioscler. Thromb. Vasc. Biol..

[94] Olano-Martin E., Anil E., Caslake M.J., Packard C.J., Bedford D., Stewart G., Peiris D., Williams C.M., Minihane A.M. (2010). Contribution of apolipoprotein E genotype and docosahexaenoic acid to the LDL-cholesterol response to fish oil. Atherosclerosis.

[95] Plourde M., Vohl M.C., Vandal M., Couture P., Lemieux S., Cunnane S.C. (2009). Plasma *n*-3 fatty acid response to an *n*-3 fatty acid supplement is modulated by APOE epsilon4 but not by the common PPAR-alpha L162V polymorphism in men. Br. J. Nutr..

[96] Chouinard-Watkins R., Rioux-Perreault C., Fortier M., Tremblay-Mercier J., Zhang Y., Lawrence P., Vohl M.C., Perron P., Lorrain D., Brenna J.T. (2013). Disturbance in uniformly ^13^C-labelled DHA metabolism in elderly human subjects carrying the APOE epsilon4 allele. Br. J. Nutr..

[97] Plourde M., Chouinard-Watkins R., Rioux-Perreault C., Fortier M., Dang M.T., Allard M.J., Tremblay-Mercier J., Zhang Y., Lawrence P., Vohl M.C. (2014). Kinetics of ^13^C-DHA before and during fish-oil supplementation in healthy older individuals. Am. J. Clin. Nutr..

[98] Conway V., Allard M.J., Minihane A.M., Jackson K.G., Lovegrove J.A., Plourde M. (2014). Postprandial enrichment of triacylglycerol-rich lipoproteins with omega-3 fatty acids: Lack of an interaction with apolipoprotein E genotype?. Lipids Health Dis..

[99] Dang T.M., Conway V., Plourde M. Disrupt Fatty Acid Distribution in HDL and LDL According to Apolipoprotein E Genotype. Proceedings of the 7th Congress of the International Society of Nutrigenetics/Nutrigenomics (ISNN).

[100] Gregg R.E., Zech L.A., Schaefer E.J., Stark D., Wilson D., Brewer H.B. (1986). Abnormal *in vivo* metabolism of apolipoprotein E4 in humans. J. Clin. Investig..

[101] Weisgraber K.H. (1994). Apolipoprotein E: Structure-function relationships. Adv. Protein Chem..

[102] Bour A., Grootendorst J., Vogel E., Kelche C., Dodart J.C., Bales K., Moreau P.H., Sullivan P.M., Mathis C. (2008). Middle-aged human APOE4 targeted-replacement mice show retention deficits on a wide range of spatial memory tasks. Behav. Brain Res..

[103] Siegel J.A., Haley G.E., Raber J. (2010). Apolipoprotein E isoform-dependent effects on anxiety and cognition in female TR mice. Neurobiol. Aging.

[104] Bourre J.M. (2004). Roles of unsaturated fatty acids (especially omega-3 fatty acids) in the brain at various ages and during ageing. J. Nutr. Health Aging.

[105] Klein R.C., Mace B.E., Moore S.D., Sullivan P.M. (2010). Progressive loss of synaptic integrity in human apolipoprotein E4 targeted replacement mice and attenuation by apolipoprotein E2. Neuroscience.

[106] Nishitsuji K., Hosono T., Nakamura T., Bu G., Michikawa M. (2011). Apolipoprotein E regulates the integrity of tight junctions in an isoform-dependent manner in an *in vitro* blood-brain barrier model. J. Biol. Chem..

[107] Liu Q., Trotter J., Zhang J., Peters M.M., Cheng H., Bao J., Han X., Weeber E.J., Bu G. (2010). Neuronal LRP1 knockout in adult mice leads to impaired brain lipid metabolism and progressive, age-dependent synapse loss and neurodegeneration. J. Neurosci..

[108] Bu G. (2009). Apolipoprotein E and its receptors in Alzheimer’s disease: Pathways, pathogenesis and therapy. Nat. Rev. Neurosci..

[109] Mahley R.W., Weisgraber K.H., Huang Y. (2009). Apolipoprotein E: Structure determines function, from atherosclerosis to Alzheimer’s disease to aids. J. Lipid Res..

[110] Dagenais C., Rousselle C., Pollack G.M., Scherrmann J.M. (2000). Development of an *in situ* mouse brain perfusion model and its application to mdr1a P-glycoprotein-deficient mice. J. Cereb. Blood Flow Metab..

[111] Ouellet M., Emond V., Chen C.T., Julien C., Bourasset F., Oddo S., LaFerla F., Bazinet R.P., Calon F. (2009). Diffusion of docosahexaenoic and eicosapentaenoic acids through the blood-brain barrier: An *in situ* cerebral perfusion study. Neurochem. Int..

[112] Vandal M., Alata W., Tremblay C., Rioux-Perreault C., Salem N., Calon F., Plourde M. (2014). Reduction in DHA transport to the brain of mice expressing human APOE4 compared to APOE2. J. Neurochem..

[113] Poirier J. (2008). Apolipoprotein E represents a potent gene-based therapeutic target for the treatment of sporadic Alzheimer’s disease. Alzheimers Dement..

[114] Conway V., Larouche A., Alata W., Vandal M., Calon F., Plourde M. (2014). Apolipoprotein E isoforms disrupt long-chain fatty acid distribution in the plasma, the liver and the adipose tissue of mice. Prostaglandins Leukot. Essent. Fatty Acids.

[115] Ravona-Springer R., Davidson M., Noy S. (2003). Is the distinction between Alzheimer’s disease and vascular dementia possible and relevant?. Dialogues Clin. Neurosci..

[116] Roher A.E., Debbins J.P., Malek-Ahmadi M., Chen K., Pipe J.G., Maze S., Belden C., Maarouf C.L., Thiyyagura P., Mo H. (2012). Cerebral blood flow in Alzheimer’s disease. Vasc. Health Risk Manag..

[117] Kalaria R.N., Ballard C. (1999). Overlap between pathology of Alzheimer disease and vascular dementia. Alzheimer Dis. Assoc. Disord..

[118] Norton S., Matthews F.E., Barnes D.E., Yaffe K., Brayne C. (2014). Potential for primary prevention of Alzheimer’s disease: An analysis of population-based data. Lancet Neurol..

[119] Kivipelto M., Helkala E.L., Laakso M.P., Hanninen T., Hallikainen M., Alhainen K., Soininen H., Tuomilehto J., Nissinen A. (2001). Midlife vascular risk factors and Alzheimer’s disease in later life: Longitudinal, population based study. BMJ.

[120] Notkola I.L., Sulkava R., Pekkanen J., Erkinjuntti T., Ehnholm C., Kivinen P., Tuomilehto J., Nissinen A. (1998). Serum total cholesterol, apolipoprotein E epsilon 4 allele, and Alzheimer’s disease. Neuroepidemiology.

[121] Bell R.D., Winkler E.A., Singh I., Sagare A.P., Deane R., Wu Z., Holtzman D.M., Betsholtz C., Armulik A., Sallstrom J. (2012). Apolipoprotein E controls cerebrovascular integrity via cyclophilin A. Nature.

[122] Halliday M.R., Pomara N., Sagare A.P., Mack W.J., Frangione B., Zlokovic B.V. (2013). Relationship between cyclophilin A levels and matrix metalloproteinase 9 activity in cerebrospinal fluid of cognitively normal apolipoprotein E4 carriers and blood-brain barrier breakdown. JAMA Neurol..

[123] Vandal M., Freemantle E., Tremblay-Mercier J., Plourde M., Fortier M., Bruneau J., Gagnon J., Begin M., Cunnane S.C. (2008). Plasma omega-3 fatty acid response to a fish oil supplement in the healthy elderly. Lipids.

[124] Plourde M., Tremblay-Mercier J., Fortier M., Pifferi F., Cunnane S.C. (2009). Eicosapentaenoic acid decreases postprandial beta-hydroxybutyrate and free fatty acid responses in healthy young and elderly. Nutrition.

[125] Fanelli F., Sepe S., D’Amelio M., Bernardi C., Cristiano L., Cimini A., Cecconi F., Ceru M.P., Moreno S. (2013). Age-dependent roles of peroxisomes in the hippocampus of a transgenic mouse model of Alzheimer’s disease. Mol. Neurodegener..

[126] Shi R., Zhang Y., Shi Y., Shi S., Jiang L. (2012). Inhibition of peroxisomal β-oxidation by thioridazine increases the amount of VLCFAs and Aβ generation in the rat brain. Neurosci. Lett..

[127] Astarita G., Jung K.M., Berchtold N.C., Nguyen V.Q., Gillen D.L., Head E., Cotman C.W., Piomelli D. (2010). Deficient liver biosynthesis of docosahexaenoic acid correlates with cognitive impairment in Alzheimer’s disease. PLoS One.

[128] Lizard G., Rouaud O., Demarquoy J., Cherkaoui-Malki M., Iuliano L. (2012). Potential roles of peroxisomes in Alzheimer’s disease and in dementia of the Alzheimer’s type. J. Alzheimer’s Dis..

[129] Guzzardi M.A., Iozzo P. (2011). Fatty heart, cardiac damage, and inflammation. Rev. Diabet. Stud..

[130] Martin M.A., Gomez M.A., Guillen F., Bornstein B., Campos Y., Rubio J.C., de la Calzada C.S., Arenas J. (2000). Myocardial carnitine and carnitine palmitoyltransferase deficiencies in patients with severe heart failure. Biochim. Biophys. Acta.

[131] Ben-Zvi A., Lacoste B., Kur E., Andreone B.J., Mayshar Y., Yan H., Gu C. (2014). Mfsd2a is critical for the formation and function of the blood-brain barrier. Nature.

[132] Nguyen L.N., Ma D., Shui G., Wong P., Cazenave-Gassiot A., Zhang X., Wenk M.R., Goh E.L., Silver D.L. (2014). Mfsd2a is a transporter for the essential omega-3 fatty acid docosahexaenoic acid. Nature.

[133] Zhao Z., Zlokovic B.V. (2014). Blood-brain barrier: A dual life of Mfsd2a?. Neuron.

[134] Betsholtz C. (2014). Physiology: Double function at the blood-brain barrier. Nature.

